# The use of end-tidal carbon dioxide monitoring in patients with hypotension in the emergency department

**DOI:** 10.1186/1865-1380-5-31

**Published:** 2012-07-24

**Authors:** Cheah P Kheng, Nik H Rahman

**Affiliations:** 1 Department of Emergency & Trauma, Hospital Queen Elizabeth, Kota Kinabalu, Sabah, Malaysia; 2 Department of Emergency Medicine, School of Medical Sciences, USM, Kota Bharu, 16150, Malaysia

**Keywords:** End-tidal carbon dioxide, Shock, Hypotension, Emergency department

## Abstract

**Background:**

The aim of this study was to determine the usefulness of end tidal carbon dioxide (ETCO_2_) monitoring in hypotensive shock patients presenting to the ED.

**Methods:**

This was a prospective observational study in a tertiary ED. One hundred three adults in shock with hypotension presenting to the ED were recruited into the study. They were grouped according to different types of shock, hypovolemic, cardiogenic, septic and others. Vital signs and ETCO_2_ were measured on presentation and at 30-min intervals up to 120 min. Blood gases and serum lactate levels were obtained on arrival. All patients were managed according to standard protocols and treatment regimes. Patient survival up to hospital admission and at 30 days was recorded.

**Results:**

Mean ETCO_2_ for all patients on arrival was 29.07 ± 9.96 mmHg. Average ETCO_2_ for patients in hypovolemic, cardiogenic and septic shock was 29.64 ± 11.49, 28.60 ± 9.87 and 27.81 ± 7.39 mmHg, respectively. ETCO_2_ on arrival was positively correlated with systolic and diastolic BP, MAP, bicarbonate, base excess and lactate when analyzed in all shock patients. Early ETCO_2_ measurements were found to be significantly lower in patients who did not survive to hospital admission (*p* = 0.005). All patients who had ETCO_2_ ≤ 12mmHg died in the ED. However, normal ETCO_2_ does not ensure patient survival.

**Conclusion:**

The use of ETCO_2_ in the ED has great potential to be used as a method of non-invasive monitoring of patients in shock.

## Background

Shock with hypotension is the main presentation of many diseases to the emergency department (ED). Patients can present with hypovolemic, cardiogenic, anaphylactic, neurogenic or even septicemic shock. The presentation of shock may be obvious as in a patient with the ultimate shock state, cardiac arrest or mildly as in the patient with decompensated cardiac failure. With the evolving treatment and management of shock, the mortality remains high. Emergency physicians and scientists are continuously trying to find new methods to recognize shock at an earlier stage and initiate early treatment [1]. Traditionally, initial therapy of shock in the emergency department concentrates on the normalization of vital signs such as heart rate, mean arterial pressure (MAP) and central venous pressure rather than restoration of adequate tissue oxygenation and perfusion. This concept is not reliable as blood flow cannot be reliably inferred from heart rate and blood pressure measurements until extreme hypotension occurs [2].

Capnography has been used extensively during cardiac arrest situations when there is a need to do CPR. Cardiac arrest diminishes cardiac output and therefore decreases elimination of carbon dioxide (CO_2_) from the lungs. Successful resuscitation will show an increase in end tidal carbon dioxide (ETCO_2_). It has helped as a prognostic tool and has even been suggested to be used as a marker of futility [3]. ETCO_2_ has been well documented to be reduced in volume-related hypotensive states where the cardiac output is reduced. In this study, we would like to investigate the usefulness of this tool in the ED particularly in patients with shock [4,5]. We attempted a study to determine the usefulness of ETCO_2_ in patients with shock who present to the ED. In specific, we would like to determine the correlation between ETCO_2_ and traditional vital signs and laboratory findings, and to compare early mean ETCO_2_ with outcome of patients in shock.

## Methods

**Figure 1:**
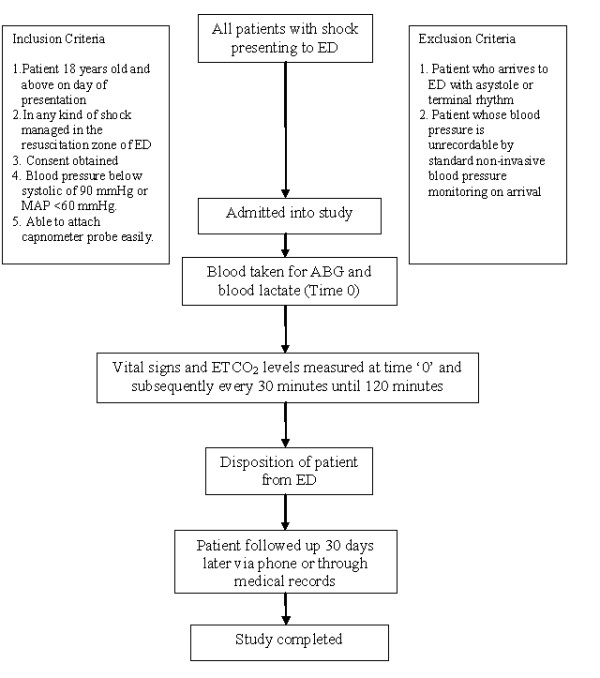
Flowchart of the study.

This prospective observational study was conducted in a regional tertiary referral center with annual patient attendance to ED of approximately 60,000. It is a teaching hospital for undergraduate and residency-based programs with many specialized fields including Emergency Medicine. All patients who presented to the ED from 1 June 2009 to 30 December 2009 with clinical signs of shock managed in the resuscitation zone were enrolled in this study. No randomization was done. Consent was obtained from either the patient or next of kin by signing the consent form (Figure 1).

The study proposal was presented to the department board review and the ethics committee. Approval was obtained from the university research ethics committee (Human) on 16 March 2009,reference no. USMKK/PPP/JEPeM [200.4(1.7)]. The study was funded by using the short-term grant (1-year duration) provided by the university (grant no. 1001/PPSP/812091). The makers of the equipment used throughout the study had no involvement. The researchers declared no conflict of interest.

Inclusion and exclusion criteria were as follows:

Inclusion criteria:

(1) Patient aged 18 years old and above on the day of presentation

(2) Be in any kind of shock state managed in the ED

(3) Initial blood pressure below systolic of 90 mmHg or MAP < 60 mmHg.

(4) Able to attach the capnometer probe easily.

Exclusion criteria:

(1) Patient who arrives to ED in asystole or in a terminal rhythm.

(2) Patient whose blood pressure is unrecordable by standard non-invasive blood pressure monitoring on arrival.

(3) Patients who had received resuscitation in the primary health center prior to transportation to the study center.

(4) Patients who are end-of-life, terminally ill and have advanced directives for do not resuscitate or attempt of active resuscitation.

(5) Elevated [P(a-ETCO_2_)] gradient of more than 6 mmHg suggesting the presence of a complex pulmonary pathology that affects the ETCO_2_

### Sample size calculation

#### To compare means of early ETCO_2_ with outcome of patients in shock

Software: PS

Independent *t*-test calculation

Power: 80%

Type 1 error: 5%

Diff: 10

Within SD: 14.7mmHg (Asplin and White, 1995) [6]

Ratio: 3 (alive)/ 1 (dead)

### Variables

Independent variable

(1) ETCO_2_

Dependent variables

(1) Blood pressure, MAP, heart rate, respiratory rate, oxygen saturation at time 0, 30min, 60min, 90min and 120min after arrival

(2) Blood lactate, pH, P_a_CO_2_, base excess and bicarbonate levels on arrival

(3) Diagnosis and classification to a specific type of shock

(4) Immediate survival to hospital admission

(5) Short-term survival at 30 days

### Preparation

Briefing sessions were given to staff working in the ED. Emergency medicine residents were informed regarding the inclusion and exclusion criteria, data collection procedures and consent, and also familiarized with the data collection sheets. Staff nurses in the ED were trained to use the ETCO_2_ sampling device for intubated and non-intubated patients. Proper reading procedure was also emphasized for accuracy of reading.

### Equipments

The Datascope® Passport 2 monitoring system, manufactured in the USA, was used for this study. This monitoring device includes noninvasive blood pressure, pulse rate, respiratory rate, O_2_ saturation and ETCO_2_. ETCO_2_ readings were obtained through the Microstream® ETCO_2_ technology by using the FilterLine™ set line for intubated patients and the CapnoLine™ sets for non-intubated patients. ETCO_2_ monitoring for non-intubated spontaneously breathing patients was achieved through the nasal cannula device. Expiratory air from the patient was sampled by the set and streamed to the monitor where the ETCO_2_ reading and capnograph were displayed. Failure of the tracing to achieve a true plateau can possibly occur under circumstances of inappropriate sampling technique, brief exhalation or heterogeneously distributed ventilation. Nurses were trained to take a reading only when the normal waveform is seen on the monitor. This ensured accuracy of the reading. The ETCO_2_ function of the machine was calibrated daily according to the manufacturer recommendation.

### Data entry

Data entry was done using the data collection sheet. Data collected include the patient’s registration number, sex, age, ethnic group, past medical history and current diagnosis. The most likely type of shock was recorded, and allowance was given to record more than one type of shock depending on patient’s presentation.

### Data analysis

All data collected were computed and analyzed using the Statistical Packages for Social Science (SPSS) by SPSS Inc., Chicago Ill., software version 12.0.1, registered to the medical school. Independent *t*-test and Pearson’s correlation were used in this study.

## Results

This study was carried out over a period of 7 months, from 1 June 2009 until 30 December 2009. A total of 103 patients were successfully enrolled in this study. Fifty-two percent (*n* = 54) were male patients with mean age for the whole study group of 54 years (SD ± 17.58). We had eliminated nine patients with the [P(a-ETCO_2_)] of more than 6 mmHg from the study as this value indicated patients might have more than one pulmonary pathology, which would affect the accuracy of ETCO_2_ interpretation. The types of shock presented were hypovolemic shock (*n* = 37), septic shock (*n* = 34) and cardiogenic shock (*n* = 30). Only two cases presented with another type of shock, namely anaphylactic shock. The mean end tidal CO_2_ on arrival in the department was 29.07 mmHg (SD ± 9.96). Endotracheal intubation was carried out in 48% (*n* = 49) of the studied sample.

**Figure 2:**
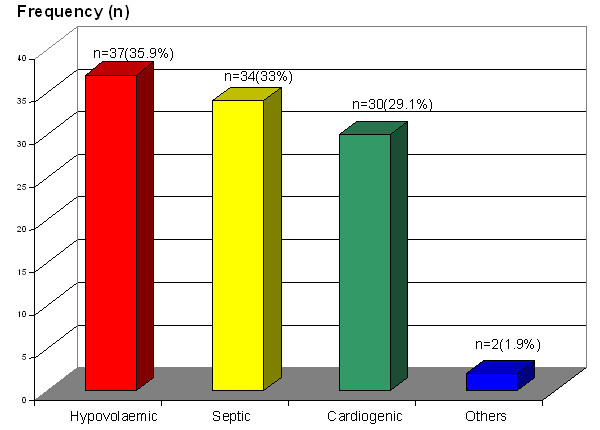
Distribution of type of shock among the study population.

**Figure 3:**
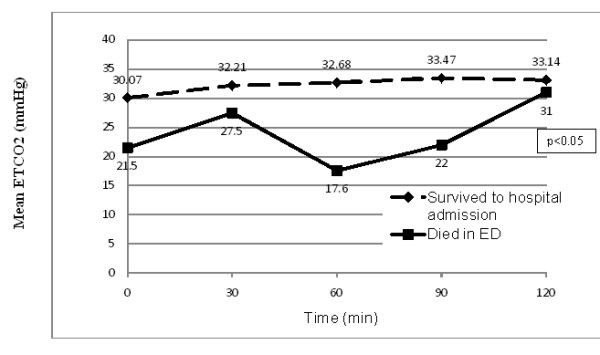
**Values of ETCO**_**2**_**for both early mortality and survival to hospital admission.**.

The majority of study participants presented with only one type of shock, with only 13 patients (12.6%) having a diagnosis of two types of shock on arrival. Of these mixed shock presentations, seven had mixed septic and hypovolemic shock (*n* = 7), and others were mixed cardiogenic and septic shock (*n* = 6). Distribution of the types of shock on presentation was also quite balanced, with a slight majority of patients presenting with hypovolemic shock (35.9%). However, there were only two patients who presented with shock other than hypovolemic, septic or cardiogenic; hence, they were grouped as others (Figure 2). We found that almost half of the study subjects needed airway intubation in the ED. The comparison of the vital parameters at 0 and 120 min after arrival in the ED in both sexes is as shown in Table 1. The intubated patients were more likely to die in the ED with a survival rate of just 75.5% as compared to a 100% survival of non-intubated patients. The ETCO_2_ levels between patients who survived to hospital admission and those who died in the ED were significant at zero min (*p* = 0.005). Early measurements of diastolic BP, bicarbonate, base excess and blood lactate also showed a significant difference between immediate and 30-day survivors and non-survivors to hospital admission (Figure 3 and Table 2).

**Table 1:** **Characteristics of male and female respondents by independent** ***t*-test**

	***Male***	***Female***	***t*** **Statistic** ***(df)***	***p*** **Value**	***95% Confidence interval***	
	**Mean ± SD**	**Mean ± SD**			**Lower**	**Upper**
Age	52.4 ± 17.84	55.90 ± 17.28	1.007(101)	0.317	−3.39	10.37
ETCO_2_ at 0 min(mmHg)	28.48 ± 9.91	29.72 ± 10.07	0.631(101)	0.53	−2.67	5.15
ETCO_2_ at 120 min(mmHg)	31.94 ± 8.30	34.18 ± 8.89	1.207(84)	0.231	−4.45	5.93
Lactate at 0 min (mmol/l)	3.72 ± 3.17	3.46 ± 2.58	−0.449(101)	0.654	−1.39	0.88
Lactate at 120 min (mmol/l)	2.81 ± 2.84	2.89 ± 2.44	0.35(70)	0.972	−1.08	1.12
Systolic BP at 0 min (mmHG)	80.33 ± 13.00	78.18 ± 9.30	−0.956	0.341	−6.61	2.31
Systolic BP at 120 min (mmHG)	116.26 ± 15.29	107.25 ± 18.59	−2.449(84)	0.016*	−16.33	−1.70
**Diastolic BP at 0 min (mmHG)**	**47.93** ± 11.20	48.91 ± 12.88	0.414(101)	0.680	−3.72	5.69
Diastolic BP at 120 min (mmHG)	66.62 ± 7.21	62.21 ± 10.02	−2.352(78.18)	0.021*	−8.15	−0.68
MAP at 0 min (mmHG)	58.64 ± 12.72	59.49 ± 10.16	0.379(99.44)	0.705	−3.62	5.34
MAP at 120 min (mmHG)	84.82 ± 7.92	77.48 ± 12.79	−3.215(84)	0.002*	−11.89	−2.78

**Table 2:** Correlation of various parameters with 30-day mortality

***Variables at 0 min***	***Median (interquartile range)***		***z Statistic***	***p Value***
	**Alive at 30 days (*n* = 27)**	**Dead at 30 days (*n* = 25)**		
Age (years)	54(34.0)	57(21.5)	−1.301	0.193
ETCO_2_ (mmHg)	32(8.5)	25(18.5)	−3.653	<0.001
Systolic BP (mmHg)	81(16.0)	85(18.5)	−1.066	0.287
Diastolic BP (mmHg)	40(22.0)	47(21.5)	−0.340	0.734
MAP (mmHg)	62(18.0)	52(23.0)	−0.690	0.490
Pulse rate (beat/min)	105(27.0)	112(34.0)	−0.937	0.876
Respiratory rate (breaths/min)	22(12.0)	26(10.0)	−0.937	0.349
SpO_2_(%)	100(2.0)	97(10.0)	−3.023	**0.002**
PaCO_2_(mmHg)	128(64.9)	117(136.7)	−0.046	0.963
Blood pH	7.45(0.32)	7.37(0.19)	−0.889	0.374
PaCO_2_(mmHg)	30.7(10.0)	30.5(15.7)	−0.687	0.492
Bicarbonate (mmol/l)	22.4(6.3)	18.6(8.75)	−2.208	**0.027**
Base excess (mmol/l)	−3.2(6.4)	−9.5(10.0)	−2.593	**0.010**
Blood lactate (mmol/l)	1.3(1.2)	3.3(5.1)	−4.778	**<0.001**

Mann-Whitney U test.

## Discussion

Capnography has been used extensively during cardiac arrest situations when there is a need to do cardiopulmonary resuscitation. Cardiac arrest and shock states diminish cardiac output and therefore decrease elimination of carbon dioxide (CO_2_) from the lungs. Successful resuscitation will show an increase in end tidal carbon dioxide (ETCO_2_). It has helped as a prognostic tool and its use has even been suggested as a marker of futility [7]. Cardiac output is affected by preload, afterload, rate, rhythm, contractility and the presence of shunt. Monitoring of these parameters will be helpful in determining the appropriate intervention to alter cardiac output. An early study in pentobarbital-anesthetized dogs explains the physiologic response of ETCO_2_ levels. When cardiac output was reduced, CO_2_ delivery to the lungs decreased, thus causing a reduction of alveolar PCO_2_ causing part of the decrease in ETCO_2_. An increase in alveolar dead space causes the remaining reduction in ETCO_2_. The lower pulmonary perfusion resulting from the decrease in cardiac output dilutes the CO_2_ in the perfused alveolar spaces, reducing ETCO_2_ even further. When the reduction of cardiac output was sustained, CO_2_ accumulation in the peripheral tissues and in venous blood increased and began to restore CO_2_ delivery to the lung, bringing ETCO_2_ levels towards baseline.

ETCO_2_ is used to monitor patients in numerous critically ill conditions. It is traditionally used in intensive care units and operation theatres, and more recently in the pre-hospital care setting and also in the ED. Other investigated indicators that have proved to be promising in the treatment of shock are blood lactate, base deficit, gastric mucosal pH, tissue oxygen concentration and venous hypercarbia. Currently, blood lactate has been shown to have good predictive value of mortality, assessing response to resuscitation and in the assessment of severity. There has also been evidence of ETCO_2_ being a predictor of high blood lactate levels [8]. The ETCO_2_ should be able to reflect any systemic hypoperfusion non-invasively and almost immediately. However, as mentioned previously, ETCO_2_ is affected by cardiac output because cardiac output is the delivery system of CO_2_ to the lung alveoli. With decreased cardiac output and constant alveolar ventilation, ETCO_2_ levels would instead be low in situations of low cardiac output but stabilize at a different level if the situation is persistent [9]. This relationship in shock patients is complicated and currently no absolute levels of ETCO_2_ are available for reference. Exceptions such as pulmonary diseases must be taken into consideration as well as the decrease of cardiac output and pulmonary blood flow,which might alter the ETCO_2_ concentration.

In our study, average ETCO_2_ at 0 min was 29.07 ± 9.96mmHg (range from 11 to 62 mmHg). It was noted that 73.8% (*n* = 76) of patients had an abnormal initial ETCO_2_ reading of below 35mmHg, which is the lower limit of the normal range. The lowest ETCO_2_ reading obtained was zero. When investigated further, all patients who had zero as their ETCO_2_ reading in the data collection sheet had actually died immediately in the ED after the reading was taken. Our finding of low ETCO_2_ levels in our patient group was not surprising as most of the patients were critically ill. Although there is no study looking at the same type of patient group for comparison, we can perhaps compare almost similar groups. A 2006 Korean paper by Moon and colleagues to determine prognostic factors of hospital survival in patients resuscitated from cardiac arrest in the ED had a patient group almost similarto ours [10].

In early shock, ETCO_2_ is expected to drop because of the reduction of cardiac output. In our study, non-survivors to hospital admission had significantly lower mean ETCO_2_ than survivors at o min. In another study, Domsky and co-workers reported that in a group of 100 critically ill patients in surgery in the emergency operating theatre, the mortality rate for an ETCO_2_ ≤ 28mmHg was 55% compared to a lower mortality of 17% if the ETCO_2_ was >28mmHg. Their overall mortality rate in those trauma patients was 41% [11]. Although none of the above studies was performed in a similar group of patients, they do support that ETCO_2_ provides some reflection of the cardiac output and cardiac function in patients that are critically ill and can be used as a prognostic tool. A further observation from our study was that if a patient had an ETCO_2_ ≤ 12mmHg at any time during the first 120 min of arrival to the ED, the patient would not survive to hospital admission. Studies in the late 1980s already established that ETCO_2_ levels below 10mmHg are incompatible with life [12,13]. In a particular study in 1995 done in the pre-hospital setting, patients in PEA (pulseless electrical activity) were found to have an average ETCO_2_ of 3.9 ± 2.8 mmHg after 20 min of unsuccessful CPR. Although there is an indication that a low ETCO_2_indicates a grave outcome in the ED, it does not give comfort and reassurance when it is normal [14,15].

### Limitations

In this study, we selected all patients in shock that satisfy the criteria of hypotension. We knew that patients in shock might not have hypotension at the outset but may develop hypotension later. By using hypotension as criteria, we have eliminated quite a number of patients in shock, and our results might not be representative of all patients in shock. However, this would not defeat the purpose of the study,which was to use ETCO_2_ for the monitoring of patients in shock with the presence of hypotension. Issues regarding ETCO_2_ monitoring include the two types of monitoring device available. One is the nasal cannula for spontaneously breathing patients, and the other is attached to the ETT for intubated patients. In intubated patients, getting a proper capnogram on the monitor is usually easy, but the same cannot be said for the nasal cannula. Staff in the ED needs to be well trained to attach the cannula properly and view the proper capnogram before taking a reading in order for the ETCO_2_ measurement to be accurate. The time of intubation and the complications of an advanced airway were not taken into consideration in this study. Complications such as aspiration, one lung ventilation and a pneumothorax caused by intubation will affect the respiratory system and might cause respiratory failure on top of the initial diagnosis on presentation. For example, instead of a single pathology such as hypovolemic shock from an intra-abdominal injury, the patient now has two pathologies, one caused by the intubation procedure. In these cases, calculating the PaCO_2_ and ETCO_2_ gradient [P(a-ETCO_2_)] might be useful as an indicator of dead space ventilation and the changes in airway physiology.

## Conclusion

The monitoring of ETCO_2_ in ED is a useful non-invasive way to gain extra information about the patient in shock. It may also be able to guide us in making decisions regarding resuscitation and futility. However, the interpretation of ETCO_2_ in critically ill patients must be done with caution, and many other factors may affect the reading. ETCO_2_ has great potential in the field of critical care and emergency medicine as a method of noninvasive monitoring.

## Competing interests

The authors declare that they have no competing interest.

## Authors’ contributions

NHR: Proposal of study, statistics, manuscript preparation. CPK: Methodology, patient recruitment, writing up results, tabulation. All authors read and approved the final manuscript.
